# Targeted therapy with nanatinostat and valganciclovir in recurrent EBV-positive lymphoid malignancies: a phase 1b/2 study

**DOI:** 10.1182/bloodadvances.2023010330

**Published:** 2023-08-04

**Authors:** Bradley Haverkos, Onder Alpdogan, Robert Baiocchi, Jonathan E. Brammer, Tatyana A. Feldman, Marcelo Capra, Elizabeth A. Brem, Santosh Nair, Phillip Scheinberg, Juliana Pereira, Leyla Shune, Erel Joffe, Patricia Young, Susan Spruill, Afton Katkov, Robert McRae, Ivor Royston, Douglas V. Faller, Lisa Rojkjaer, Pierluigi Porcu

**Affiliations:** 1 Division of Hematology, University of Colorado, Denver, CO; 2 Division of Hematologic Malignancies and Hematopoetic Stem Cell Transplantation, Department of Medical Oncology, Thomas Jefferson University Hospital, Philadelphia, PA; 3 The Ohio State University James Comprehensive Cancer Center, Columbus, OH; 4 John Theurer Cancer Center, Hackensack University Medical Center, Hackensack, NJ; 5 Centro Integrado de Hematologia e Oncologia - Hospital Mãe de Deus, Porto Alegre, Brazil; 6 Division of Hematology/Oncology, Deptartment of Medicine, University of California, Irvine, Orange, CA; 7 Mid Florida Hematology and Oncology Center, Orange City, FL; 8 Division of Hematology, Hospital A Beneficência Portuguesa, São Paulo, Brazil; 9 Division of Hematology, Hospital das Clínicas da Faculdade de Medicina, Universidade de São Paulo, São Paulo, Brazil; 10 University of Kansas Cancer Center, University of Kansas Medical Center, Kansas City, KS; 11 Memorial Sloan Kettering Cancer Center, New York, NY; 12 Ronald Reagan UCLA Medical Center, Los Angeles, CA; 13 Applied Statistics and Consulting, Spruce Pine, NC; 14 Viracta Therapeutics, Inc, Cardiff, CA

## Abstract

Lymphomas are not infrequently associated with the Epstein-Barr virus (EBV), and EBV positivity is linked to worse outcomes in several subtypes. Nanatinostat is a class-I selective oral histone deacetylase inhibitor that induces the expression of lytic EBV BGLF4 protein kinase in EBV^+^ tumor cells, activating ganciclovir via phosphorylation, resulting in tumor cell apoptosis. This phase 1b/2 study investigated the combination of nanatinostat with valganciclovir in patients aged ≥18 years with EBV^+^ lymphomas relapsed/refractory to ≥1 prior systemic therapy with no viable curative treatment options. In the phase 1b part, 25 patients were enrolled into 5 dose escalation cohorts to determine the recommended phase 2 dose (RP2D) for phase 2 expansion. Phase 2 patients (n = 30) received RP2D (nanatinostat 20 mg daily, 4 days per week with valganciclovir 900 mg orally daily) for 28-day cycles. The primary end points were safety, RP2D determination (phase 1b), and overall response rate (ORR; phase 2). Overall, 55 patients were enrolled (B–non-Hodgkin lymphoma [B-NHL], [n = 10]; angioimmunoblastic T-cell lymphoma-NHL, [n = 21]; classical Hodgkin lymphoma, [n = 11]; and immunodeficiency-associated lymphoproliferative disorders, [n = 13]). The ORR was 40% in 43 evaluable patients (complete response rate [CRR], 19% [n = 8]) with a median duration of response of 10.4 months. For angioimmunoblastic T-cell lymphoma-NHL (n = 15; all refractory to the last prior therapy), the ORR/CRR ratio was 60%/27%. The most common adverse events were nausea (38% any grade) and cytopenia (grade 3/4 neutropenia [29%], thrombocytopenia [20%], and anemia [20%]). This novel oral regimen provided encouraging efficacy across several EBV^+^ lymphoma subtypes and warrants further evaluation; a confirmatory phase 2 study (NCT05011058) is underway. This phase 1b/2 study is registered at www.clinicaltrials.gov as #NCT03397706.

## Key Points

• Nanatinostat plus valganciclovir is a novel oral regimen for relapsed/refractory EBV^+^ lymphoma that warrants further investigation.
• Encouraging efficacy and safety were observed across a variety of EBV^+^ lymphoma subtypes.

## Visual Abstract

**Figure:**
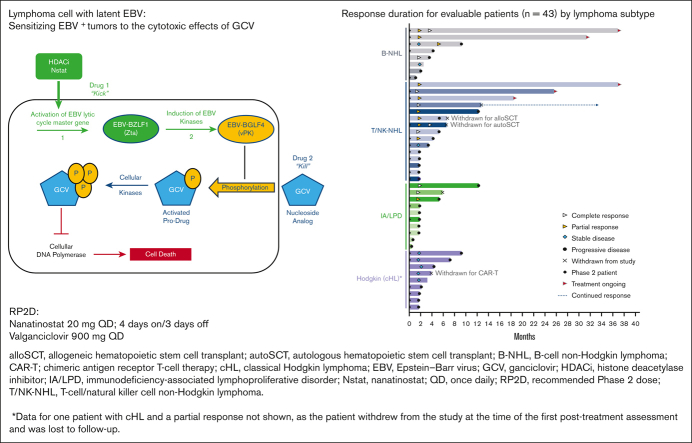

## Introduction

Epstein-Barr virus (EBV) is a human γ-herpesvirus that establishes long-term latent infection in over 90% of the adult population worldwide and is a causal factor for many lymphoid and epithelial cancers in both immunodeficient and immunocompetent patients.^1^^,^^2^ EBV is classified by the World Health Organization as a class I carcinogen^3^ and fulfills multiple criteria as a cancer driver in cancer hallmark analysis.^4^ EBV is estimated to be responsible for 1% to 2% of all human cancers, with more than 200 000 attributable cancers and 140 000 cancer-related deaths each year globally.^5^, ^6^, ^7^ EBV-positive (EBV^+^) lymphomas are a heterogeneous group of malignancies harboring latent EBV within lymphoma cells and are associated with variable clinical features and outcomes.^5^^,^^8^, ^9^, ^10^

EBV^+^ lymphomas, commonly defined by the detection of EBV-encoded RNAs (EBERs) in tumor tissues, are often aggressive and respond poorly to conventional treatments.^11^, ^12^, ^13^, ^14^, ^15^ EBV is associated with several lymphoma subtypes, with reported frequencies of 5% to 14% in diffuse large B-cell lymphoma (DLBCL), 30% to 100% in T-cell and NK-cell lymphomas, 60% to 80% in posttransplant lymphoproliferative disease (PTLD), and 15% to 30% in classical Hodgkin lymphoma (cHL).^16^ Although the contribution of EBV to lymphoma development is likely multifactorial and nuanced in immunodeficient vs immunocompetent patients and in different lymphoma subtypes,^17^^,^^18^ both retrospective and prospective data sets link EBV to inferior survival in DLBCL,^15^^,^^19^, ^20^, ^21^, ^22^ peripheral T-cell lymphoma (PTCL),^14^^,^^23^ and cHL.^24^ Most EBV^+^ lymphomas occur in immunocompetent patients and are aggressive, with a dismal prognosis and 5-year overall survival (OS) rates as low as 20% to 25% (PTCL^23^ and DLBCL not otherwise specified [NOS]^12^) and a 2-year OS of 44% (extranodal natural killer/T-cell lymphoma [ENKTL]^25^). Distinct molecular, immunological, and clinical features of EBV^+^ DLBCL and PTCL have been confirmed globally.^26^^,^^27^ Therefore, treatment approaches targeting EBV address a well-recognized unmet medical need in this high-risk population.

EBV offers an attractive, druggable nonhost target, but is predominantly latent in infected tumor cells, and most of the ∼80 protein-coding viral genes are epigenetically silenced, except for a small subset that is necessary for the maintenance and replication of the episome (EBNA1), to drive host cell proliferation, and to block apoptotic pathways (LMP-1, LMP-2A).^28^^,^^29^ Activation of the EBV lytic cycle makes EBV^+^ tumor cells vulnerable to ganciclovir (GCV), a deoxynucleoside analog that is activated by EBV kinases and inhibits both viral and cellular DNA polymerases, efficiently inducing tumor cell apoptosis in in vitro models.^30^, ^31^, ^32^, ^33^, ^34^ A small phase 1 proof-of-principle study with the pan-histone deacetylase inhibitor (HDACi) arginine butyrate and intravenous GCV demonstrated encouraging safety and efficacy, with 4 complete and 6 partial responses (PR) reported in 15 patients with refractory EBV^+^ lymphomas.^35^ However, arginine butyrate has suboptimal pharmacokinetic properties and requires prolonged intravenous infusion to maintain its therapeutic levels.^35^ GCV-containing combination treatment was also associated with a high rate of response in patients with lytic-phase protein-expressing EBV^+^ primary central nervous system PTLD.^36^

Nanatinostat (VRx-3996; CHR-3996; Nstat) is a potent, orally administered hydroxamic acid-based class I-selective HDACi highly selective for HDAC 1 to 3, with a single-agent maximum tolerated dose of 80 mg per day in patients with advanced solid tumors and a recommended phase 2 dose (RP2D) of 40 mg/d.^37^ The most common treatment-related adverse events associated with Nstat (>20%) were primarily grade 1 to 2 fatigue, nausea, constipation, anorexia, and vomiting.^37^ Nstat had a generally proportional area under the time–concentration curve, with a median half-life (t_1/2_) of 1.8 hours and a median T_max_ of 1 hour, indicating rapid absorption following oral dosing.^37^ Nstat induces lytic cycle activation in EBV-infected Burkitt’s lymphoma cells in vitro at nanomolar concentrations (unpublished data). Therefore, there exists the possibility of an innovative targeted oral therapy approach comprising Nstat to induce lytic-phase EBV protein expression and valganciclovir (VGCV), an orally bioavailable prodrug of GCV, to induce apoptosis in EBV^+^ lymphomas.

This international multicenter phase 1b/2 VT3996 to 201 study (NCT03397706) is the first clinical study to evaluate Nstat with VGCV in patients with EBV^+^ lymphoid malignancies. This study was designed to investigate the safety, pharmacokinetics (PK), and preliminary efficacy of Nstat combined with VGCV in patients with relapsed/refractory (R/R) EBV^+^ lymphoma after 1 or more prior therapies.

## Methods

The phase 1b study aimed to evaluate the safety and define the RP2D for Nstat in combination with VGCV. A phase 2 expansion cohort assessed the overall response rate (ORR) at RP2D. Secondary objectives included evaluation of PK parameters, time to and duration of response, progression-free survival, and OS. Plasma EBV DNA (pEBVd) levels were evaluated as an exploratory biomarker.

Eligible patients were ≥18 years of age with a R/R pathologically confirmed EBV^+^ lymphoid malignancy or lymphoproliferative disease of any histologic subtype, and absence of available therapy with a reasonable likelihood of cure or significant clinical benefit according to the investigator. The full eligibility criteria can be found in the supplemental Methods. For eligibility purposes, the EBV^+^ tumor status was defined by the local investigator according to the institution’s standard testing method. Although the definition of EBV^+^ status at enrollment was left to each site, centralized testing (NeoGenomics, Fort Myers, FL) defined EBER-ISH-evaluable as 100 viable tumor cells present per hematoxylin and eosin-stained slide (centralized testing was not performed to determine eligibility). The percentage of EBER-ISH-positivity was then calculated as the fraction of EBER-ISH-positive cells in all viable tumor cells. Patients with HIV-associated lymphomas were eligible for phase 1b but were excluded from phase 2 due to insufficient preliminary evidence of clinical benefit. Other notable exclusion criteria included antilymphoma therapy within 14 days, HDACi within 28 days, hematopoietic stem cell transplantation (SCT) or solid organ transplantation within 60 days, or known central nervous system lymphoma.

Phase 1b dose escalation used a modified 3+3 design; dose-limiting toxicities (DLTs) were assessed in an initial 3 to 4 patients at each dose level during the first 28-day cycle, with additional patients enrolled in the cohort if DLTs occurred (refer to definitions of DLTs in supplemental Methods). Cohorts were considered safe if ≤1 DLT occurred in 6 patients; ≥2 DLTs in an expanded cohort signified that the maximum tolerated dose had been exceeded, and the next lowest dose cohort was expanded.

For the first 43 patients enrolled, VGCV was given 1 hour before Nstat on cycle 1 day 1 to confirm the tolerability of the antiviral drug; thereafter, both drugs were taken simultaneously, and the final 12 patients enrolled in phase 2 took both drugs together throughout, following a protocol amendment. Nstat (5 mg capsules) and VGCV were given in 28-day cycles according to the regimens in Table 1. Treatment was continued for as long as the patients experienced a clinical benefit in the opinion of the investigator and in the absence of unacceptable toxicity. The treatment regimen was adapted between cohorts according to safety experience; Nstat and VGCV were given continuously in cohorts 1 and 2, but in cohort 3 (established as the RP2D cohort), Nstat was given on a 4 days on/3 days off cycle with continuous daily oral VGCV.^38^ After RP2D selection, 30 additional patients were enrolled in the phase 2 RP2D expansion cohort.

**Table 1.** **Phase 1b study dose cohorts (n = 25)**

Cohort	n	Nanatinostat dose	VGCV dose	Responses	DLTs
1	7∗	10 mg BID	900 mg BID1 (n = 4) 450 mg BID2 (n = 3)	2 CR, 1 PR	G4 neutropenia (n = 1) G4 thrombocytopenia (n = 1) G3 thrombocytopenia (n = 2)
2a	5†	5 mg BID	450 mg BID	1 CR, 1 PR	No DLT
2b	4∗	10 mg QD	450 mg BID	1 CR, 1 PR	No DLT
2c	4	10 mg QD	900 mg QD	–	No DLT
3‡	5	20 mg QD 4 days on/3 days off schedule	900 mg QD	1 CR, 2 PR	No DLT

BID, twice daily; G, grade.

∗ One patient not evaluable.

† Two patients not evaluable.

‡ This regimen chosen as the RP2D; nanatinostat given on days 1 to 4, 8 to 11, 15 to 18, and 22 to 25 of each cycle.

Efficacy was assessed by the investigator using positron emission tomography–computed tomography according to the Lugano 2014 criteria.^39^ Response assessments were performed every 2 cycles until disease progression or study withdrawal. Safety was assessed by the investigator via adverse events (AEs) (NCI CTCAE version 5.0) from the first administration of the study drug until 28 days after the last dose or until the start of a new anticancer therapy. Laboratory values (hematology and biochemistry) were monitored every 2 weeks, VGCV dose adjustment guidance for creatinine clearance was followed per label, and sequential boundaries were used to monitor the DLT rate during phase 2 and reviewed by the Safety Review Committee (SRC). Blood samples for PK investigations were taken during cycle 1 on day 1 predose and at 1, 1.5, 2, 3, 5, and 7 hours after dose for VGCV when given 1 hour before Nstat (correlating to 0.5, 1, 2, 4, and 6 hours after dose for Nstat). Plasma EBV and cytomegalovirus (CMV) DNA levels were monitored by quantitative polymerase chain reaction (Eurofins Viracor, Inc, MO; lower limit of detection 38 IU/mL [plasma EBV] and 56 IU/mL [CMV]) at baseline, weekly during C1, then on day 1 of cycles 2 to 12. This study was approved by the Institutional Review Board of each site and conducted in accordance with the Declaration of Helsinki. All patients gave written informed consent.

### Statistical methods

The primary analysis was performed when all responding patients in phase 2 were followed-up for up to 12 months. The RP2D was determined by the SRC after the phase 1b study, and sequential boundaries were used to monitor the DLT rate during phase 2. The sample size calculation for phase 2 is detailed in the supplemental Methods. Descriptive statistics were calculated for all the end points. Duration of response (data cut on 1 September 2022) with an additional 10 months of follow-up is presented as an updated analysis, as primary analysis data are presented in abstract form.^40^

The safety data set included all patients who received Nstat, and the efficacy data set included all patients from phase 1 and 2 with evaluable/measurable disease and ≥1 after baseline tumor assessment.

## Results

### Patients

Fifty-five patients were enrolled at 19 centers (14 in the United States and 5 in Brazil) between March 2018 and March 2021, 25 in phase 1b, and 30 in phase 2 (supplemental Figure 1). EBV-positivity in tumor tissue was locally determined (EBER-ISH in 52 patients and LMP-1 IHC in 3 patients) and documented by the local investigator. Patient characteristics are outlined in Table 2. Most patients (38%) had angioimmunoblastic T-cell lymphoma (T/NK)-NHL (n = 21: ENKTL [n = 9], PTCL-NOS [n = 5], angioimmunoblastic T-cell lymphoma [AITL] [n = 6], CTCL [n = 1]) or immunodeficiency–associated lymphoproliferative disorders (IA-LPD) (n = 13 [24%]), with the remaining patients split between B-cell NHL (n = 10 [18%]; EBV^+^ DLBCL, NOS [n = 7] and other B-cell types [n = 3]) and cHL (n = 11 [20%]). Patients had a median age of 60 years, predominantly stage III to IV disease (84%), and were heavily pretreated, with a median of 2 prior lines of therapy (range 1-11), including brentuximab vedotin (n = 13, 24%), checkpoint inhibitors (n = 9, 16%), and HDACi (n = 5, 9%). In addition, 13 patients (24%) underwent autologous (n = 9) or allogeneic (n = 4) hematopoietic SCT, and 5 patients (9%) received EBV-targeted cytotoxic T-cell therapy. Most of the patients (75%) were refractory to the most recent prior therapy.

**Table 2.** **Patient characteristics**

	All (N = 55)	Phase 1b (n = 25)	Phase 2 (N = 30)
Median age, y (range)	60 (19-84)	58 (19-84)	67 (23-81)
Male/female, n/n	35/20	17/8	18/12
**ECOG performance status, n (%)**			
0-1	48 (87%)	23 (92%)	25 (83%)
2	7 (13%)	2 (8%)	5 (17%)
**Stage**			
I-II	9 (16%)	6 (24%)	3 (10%)
III-IV	46 (84%)	19 (76%)	27 (90%)
**Previous lines of antineoplastic therapy, n (%)**			
1	13 (24%)	5 (20%)	8 (27%)
2	19 (35%)	9 (36%)	10 (33%)
≥3	23 (42%)	11 (44%)	12 (40%)
Median, n (range)	2 (1-11)	2 (1-11)	2 (1-6)
**Prior therapies**			
Brentuximab vedotin, n (%)	13 (24%)	7 (28%)	6 (20%)
AutoSCT/alloSCT, n (%)	13 (24%)	7 (28%)	6 (20%)
Checkpoint inhibitor, n (%)	9 (16%)	5 (20%)	4 (13%)
HDACi, n (%)	5 (9%)	3 (12%)	2 (7%)
EBV CTL	5 (9%)	2 (8%)	3 (10%)
Refractory to most recent regimen, n (%)	41 (75%)	17 (68%)	24 (80%)
**EBV**^**+**^**lymphoma diagnosis**			
B-NHL, n (%)	10 (18%)		
DLBCL, n	7		
Other B-cell, n	3∗		
T/NK-NHL, n (%)	21 (38%)		
Extranodal NK/T-cell lymphoma	9		
PTCL NOS	5		
Angioimmunoblastic T-cell lymphoma	6		
Cutaneous T-cell lymphoma	1		
**Immunodeficiency-associated LPD, n (%)**	13 (24%)		
Posttransplant lymphoproliferative disorder	4		
Systemic lupus erythematosus	2		
Common variable immune deficiency	1		
Primary immunodeficiency	1		
HIV-associated lymphoma (PBL, DLBCL, HL)	5		
Hodgkin lymphoma, n (%)	11 (20%)		

alloSCT, allogeneic SCT; autoSCT, autologous SCT; CTL, cytotoxic T lymphocytes; ECOG, Eastern Cooperative Oncology Group; HL, Hodgkin lymphoma; LPD, lymphoproliferative disorder; NHL, non-Hodgkin lymphoma; NK/T, natural killer/T-cell; PBL, plasmablastic lymphoma.

∗ CD30+ B-cell lymphoma, B-LPD, and PBL.

### Phase 1b: determination of RP2D

Twenty-five patients were enrolled in phase 1b portion of the study (Table 1). In cohort 1, following hematologic DLTs observed in 3 of the first 4 patients enrolled (grade 4 neutropenia, grade 3 thrombocytopenia, and grade 4 thrombocytopenia), the VGCV dose was reduced by 50% for the next 3 patients; 1 patient subsequently had grade 3 thrombocytopenia lasting 7 days. No DLTs occurred in cohorts 2 or 3. Because of the DLT in cohort 1, PK/progressive disease data, and preclinical experience,^38^ a 4 days on/3 days off schedule was explored for Nstat in cohort 3 at 20 mg daily (with VGCV 900 mg PO once daily [QD]). No DLTs occurred and the SRC determined this to be RP2D (refer to supplemental Results for further discussion of the rationale for RP2D selection). The patients in phase 2 (n = 30) underwent RP2D.

### Safety

Nstat in combination with VGCV therapy was generally well-tolerated; the most frequent AEs (of any grade) were nausea (38%), thrombocytopenia (36%), neutropenia (35%), anemia, and constipation (both 31%) (Table 3). The most common grade 3 to 4 events were cytopenias (thrombocytopenia, neutropenia, and anemia), which were generally uncomplicated and reversible. Serious AEs occurred in 16 patients (29%), including 8 patients (27%) in phase 2. Serious AEs that occurred in more than 1 patient were febrile neutropenia, pneumonia, sepsis, and acute kidney injury (n = 2 each); only febrile neutropenia and 1 event of sepsis were considered related to the study drug.

**Table 3.** **Treatment-emergent adverse events in greater than or equal to 20% of patients**

TEAE, n (%)	Primary analysis			
	Overall population (N = 55)		Phase 2 (n = 30)	
	All grades	Grade 3-4	All grades	Grade 3-4
Nausea	21 (38.2)	2 (3.6)	11 (36.7)	2 (6.7)
Platelet count decreased	20 (36.4)	11 (20.0)	7 (23.3)	3 (10.0)
Neutrophil count decreased	19 (34.5)	16 (29.1)	9 (30.0)	7 (23.3)
Anemia	17 (30.9)	11 (20.0)	8 (26.7)	7 (23.3)
Constipation	17 (30.9)	1 (1.8)	8 (26.7)	1 (3.3)
Serum creatinine increased	14 (25.5)	0	2 (6.7)	0
Diarrhea	14 (25.5)	1 (1.8)	6 (20.0)	1 (3.3)
Fatigue	14 (25.5)	1 (1.8)	8 (26.7)	0
Decreased appetite	12 (21.8)	0	6 (20.0)	0
Vomiting	11 (20.0)	1 (1.8)	5 (16.7)	1 (3.3)

TEAE, treatment-emergent adverse event.

Dose reductions and interruptions due to treatment–related adverse events (primarily decreased platelet or neutrophil count) were reported in 14 (25%) and 16 (29%) patients, respectively. Six patients (11%) reported a treatment-emergent AE that led to permanent treatment discontinuation (nosocomial COVID-19 pneumonia, pharyngitis, decreased neutrophil count, histiocytic sarcoma, acute kidney injury, and embolism). Of these, 2 were considered to be related to the study drug (neutrophil count decreased [G3; resolved after 2 days with granulocyte colony-stimulating factor support] and [potentially fungal] pharyngitis). There were no study drug-related deaths during the treatment period.

Three of the 55 patients had detectable CMV DNA in their blood at baseline with no signs of clinical CMV infection. In all 3 cases, CMV DNA levels were undetectable after the initiation of therapy and remained undetectable. None of the 55 patients developed CMV reactivation or clinical CMV infection during the therapy.

### Efficacy

**Figure 1.**
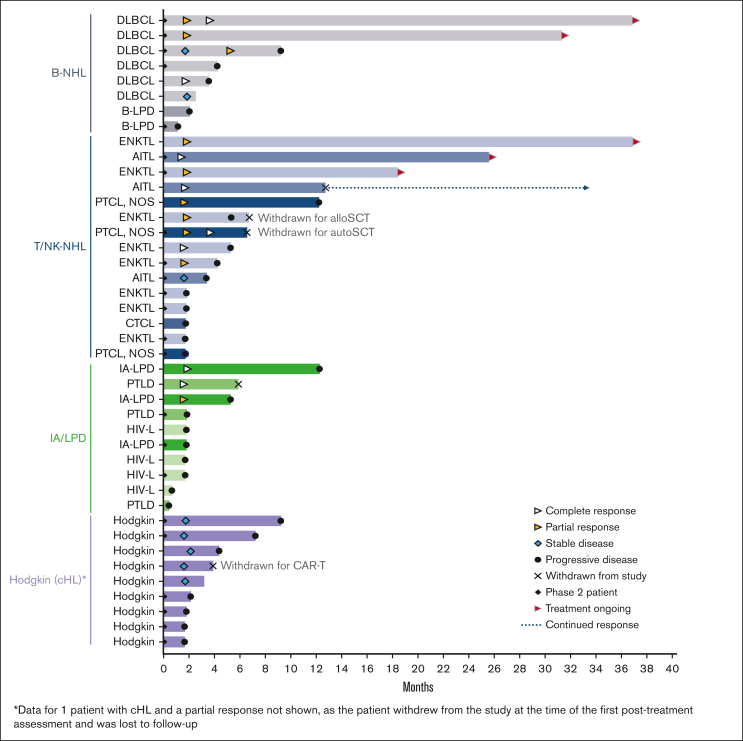
**Response duration for evaluable patients (n = 43) according to the lymphoma subtype.** AITL, angioimmunoblastic T-cell lymphoma; alloSCT, allogeneic hematopoietic stem cell transplant; autoSCT, autologous hematopoietic stem cell transplant; B-LPD, B-cell lymphoproliferative disease; B-NHL, B-cell non-Hodgkin lymphoma; CAR-T; chimeric antigen receptor T-cell therapy; cHL, classical Hodgkin lymphoma; CTCL, cutaneous T-cell lymphoma; DLBCL, diffuse large B-cell lymphoma; ENKTL, extranodal natural killer/T-cell lymphoma; HIV-L, HIV-associated lymphoma; IA-LPD, immunodeficiency-associated lymphoproliferative disorder; PTCL NOS, peripheral T-cell lymphoma not otherwise specified; PTLD, post-transplant lymphoproliferative disorder; T/NK-NHL, T-cell/natural killer cell non-Hodgkin lymphoma.

Table 4 shows responses to treatment across the 4 lymphoma categories (Table 1). Twelve patients were not evaluable for efficacy (the reasons are described in supplemental Figure 1). The ORR for 43 evaluable patients (at least 1 after treatment response assessment) was 40% (n = 17/43), with a complete response rate (CRR) of 19% (n = 8/43) and a median time to response of 1.8 months (33-162 days). All patients, except 3, achieved the best response in their first disease response assessment (Figure 1). The highest ORR and CRR were observed in patients with T/NK-NHL (n = 15; 60% and 27%, respectively); 6 had received prior SCT, 3 had received other HDACi therapy, 4 had received programmed cell death protein-1 inhibitors, and all were refractory to their most recent therapy (supplemental Table 1); one of the CRs was achieved in a patient who never responded to HDACi. Of the 6 evaluable patients with DLBCL, 2 had CR (both with primary refractory DLBCL) and 2 had PR (Table 4); one of the CRs was achieved in a patient who did not respond to first-line R-CHOP. Prior therapies and response duration in these patients are shown in supplemental Table 1.

**Table 4.** **Responses in evaluable patients (n = 43)**

Lymphoma subtype	Responses	ORR/ CR
**B-NHL (n = 8)**		
DLBCL (n = 6)	CR (2), PR (2), SD (1), PD (1)	67%/33%
Other B-cell (n = 2∗)	PD (2)	
**T/NK-NHL (n = 15)**		
Extranodal NK/T-cell lymphoma	CR (1), PR (4), PD (3)	63%/13%
PTCL, NOS	CR (1), PR (1), PD (1)	67%/50%
Angioimmunoblastic T-cell lymphoma	CR (2), SD (1)	
Cutaneous T-cell lymphoma	PD (1)	
**Immunodeficiency-associated LPD (n = 10)**		
PTLD	CR (1), PD (2)	50%/33%
Other (SLE, CVID, PI)	CR (1), PR (1), PD (1)	
HIV-associated lymphoma (PBL, DLBCL [2], HL)	PD (4)	
Hodgkin lymphoma (n = 10)	PR (1), SD (5), PD (4)	10%/0%
Total (n = 43 evaluable patients)	CR (8), PR (9), SD (7), PD (19)	40%/19%

CVID, common variable immune deficiency; HL, Hodgkin lymphoma; LPD, lymphoproliferative disorder; NHL, non-Hodgkin lymphoma; PBL, plasmablastic lymphoma; PD, progressive disease; PI, primary immunodeficiency; SD, stable disease; SLE, systemic lupus erythematosus.

∗ CD30+ B-cell lymphoma and B-LPD.

**Figure 2.**
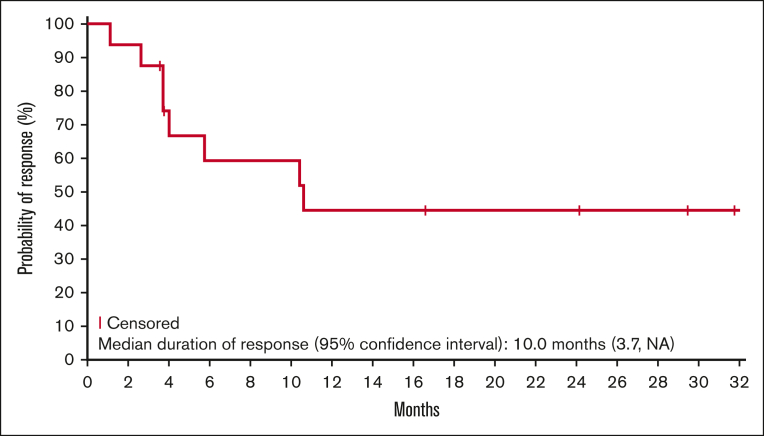
**Kaplan-Meier estimate of the duration of response (updated analysis; n = 17).**

Responses lasting ≥6 months were observed for all lymphoma types, except Hodgkin lymphoma (Figure 1). Five patients (2 DLBCL, 2 ENKTL, and 1 AITL) remained on the study treatment at the data cutoff, and 4 had ongoing responses lasting >24 months. One additional patient with AITL remained in CR 20.7 months after discontinuing therapy (supplemental Table 1). The median duration of response was 10.0 months in the updated analysis (Figure 2). Of the 17 responders, 8 had responses lasting ≥6 months.

Three patients (2 with T/NK-NHL and 1 with Hodgkin lymphoma) were withdrawn from the study after achieving a response to undergo hematopoietic cell transplantation (HCT) (1 autologous HCT and 1 allogeneic HCT) and chimeric antigen receptor (CAR) T-cell therapy.

### PK and exploratory end points

PK parameters for Nstat were derived from the individual plasma concentration-time profiles and were consistent with dose-exposure profiles from a previous phase 1 study of Nstat monotherapy in patients with solid tumors.^37^ The absorption was rapid, with a median time to maximum observed concentration (Tmax) of 1.9 hours. For the 20 mg dose, Cmax was 186 ng/mL, with a median t_½_ of 2.0 hours.

**Figure 3.**
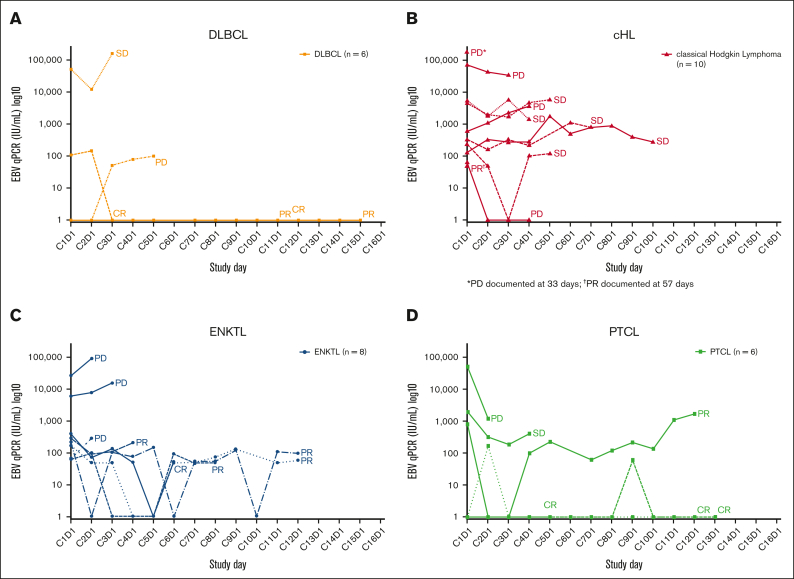
**EBV viral load during the study according to lymphoma subtype.** EBV viral load in patients with (A) DLBCL, (B) HL, (C) ENKTL, and (D) PTCL. C, cycle; cHL, classical Hodgkin lymphoma; CR, complete response; D, day; DLBCL, diffuse large B-cell lymphoma; EBV, Epstein-Barr virus; ENKTL, extranodal natural killer/T-cell lymphoma; PD, progressive disease; PR, partial response; PTCL, peripheral T-cell lymphoma; SD, stable disease.

Longitudinal pEBVd data for the lymphoma subtypes are shown in Figure 3. Baseline pEBVd values were available for 54/55 patients; overall, elevated pEBVd levels were present in 39 patients (72%). For patients with detectable viral load, the median level of pEBVd at baseline was 2200 IU/mL (range 49-575 000 IU/mL). Across lymphoma subtypes, objective responses in 7 patients with elevated pEBVd at baseline were accompanied by a decrease in EBV viral load as early as C2 and C3, whereas radiological disease progression in 7 patients was accompanied by an increase (n = 5) or no reduction (n = 2) in the level of pEBVd. In other patients, longitudinal changes in pEBVd levels did not correlate with objective response.

To explore how various criteria for EBV^+^ lymphoma may impact efficacy, we examined responses according to local (at enrollment) vs centralized (after enrollment) EBV^+^ status (supplemental Table 2). Among the 33 response-evaluable patients with centralized EBER-ISH determination, centralized estimates of EBER-ISH-positivity were available for 25 patients. In 8 of the 33 patients, centralized EBER-ISH assessment was not possible due to technical aspects (supplemental Table 3). In the remaining 25 patients, responses were observed across a broad spectrum of EBER-ISH positivity (supplemental Table 2).

## Discussion

EBV^+^ DLBCL^12^ and PTCL^41^ are increasingly being recognized as distinct subsets of aggressive, poor-risk non-Hodgkin lymphoma with unique molecular hallmarks and tumor microenvironments. The presence of EBV in DLBCL and PTCL, as in other well-known EBV^+^ tumors such as ENKTL, cHL, and IA-LPD, offers an actionable yet mostly untapped tumor-specific vulnerability. The combination of lytic cycle activators and GCV has shown antitumor activity in several preclinical models and in 1 clinical study of EBV^+^ cancers; however, clinical development has been hampered by an inconvenient dosing schedule, suboptimal PK, or low lytic activating potency. This phase 1b/2 study represents an important step forward by supporting the feasibility, safety, and preliminary efficacy of the potent oral class I-selective HDACi Nstat in combination with oral VGCV for the treatment of EBV^+^ lymphomas. The RP2D of Nstat 20 mg QD on a 4 days on/3 days off schedule, with VGCV 900 mg QD on a continuous basis, was identified and well tolerated, with the most frequent AEs being low-grade nausea, constipation, and cytopenias, which are typical for the individual drugs. Additional safety data from the RP2D expansion cohort revealed no new or unexpected safety signals. Importantly, the combination was active across a broad spectrum of EBV^+^ lymphomas, with an ORR of 40% and CRR of 19%. Patients were often refractory to their last therapy and had been exposed to multiple active prior therapies, including SCT, HDACi, brentuximab vedotin, and EBV-cytotoxic T lymphocytes, supporting the hypothesis that EBV is a shared targetable vulnerability. ORRs to single-agent HDACi in R/R DLBCL and R/R PTCL range between 5.6% and 28%^42^, ^43^, ^44^ and 25% and 29%,^45^^,^^46^ respectively, suggesting that responses, particularly in DLBCL, were not just due to Nstat. Most responses were observed at the time of the first disease response assessment, with CRs observed in DLBCL, ENKTL, PTCL, and IA-LPD. Several durable responses were observed in PTCL and DLBCL and 3 patients (PTCL, ENKTL, and HL), who at the time of enrollment were not eligible for SCT or CAR T-cell therapy, achieved adequate disease control and were able to proceed to SCT (n = 1 autologous and n = 1 allogeneic) or CAR T-cell therapy (n = 1). Therefore, Nstat and VGCV provided a novel bridging therapy to potentially curative treatment.

EBV lytic cycle activation with HDACi in combination with GCV as an effective approach to eliminate EBV-infected tumor cells has a strong mechanistic rationale and has been validated preclinically in multiple studies.^30^^,^^33^^,^^34^^,^^47^, ^48^, ^49^ Therefore, the proposed mechanism of action of the Nstat-VGCV combination in this study is Nstat-induced expression of EBV-encoded viral kinases (in particular BGLF4), followed by BLGF4-induced activation of the prodrug GCV to its monophosphorylated form. Once triphosphorylated by cellular kinases into the active drug, triphospho-GCV inhibits both viral and cellular DNA polymerases, inducing DNA chain termination, dsDNA breaks, and tumor cell apoptosis.^50^^,^^51^ Noninduced EBV^-^ tumor cells can also become targets of activated GCV via the bystander effect.^52^ Inhibition of EBV proliferation by GCV may also play a role in preventing or mitigating EBV-induced hyperprogression events, as observed in a small study of single-agent romidepsin in ENKTL.^53^ No disease flares or fulminant EBV reactivations were observed in this study, including in 9 patients with ENKTL, suggesting that the addition of VGCV to Nstat not only contributed to the efficacy of this combination but may also enhance safety through its control of viral replication. The intermittent Nstat dosing regimen also aligns with preclinical data indicating that discontinuous exposure to HDACi was sufficient to sensitize EBV^+^ Burkitt lymphoma cells (P3HR1) to GCV, introducing an opportunity to minimize potential toxicities while maintaining efficacy in clinical regimens.^33^

Although all patients enrolled in this study had EBV^+^ lymphoma, as defined locally by each site, a relationship between responses and the percentage of EBV^+^ tumor cells was not apparent in the subset in which EBER-ISH was performed centrally. However, this remains an end point for further investigation. The lack of correlation between clinical response and EBER-ISH positivity may be due to technical components, such as variability in EBER expression among tumor cells, stability of the RNA, and the efficiency of the assay to detect EBER RNA in every EBV^+^ cell. In this heterogeneous population of multiple lymphoma subtypes, EBV-based eligibility was intentionally defined in broad terms and left to each site’s determination, as there is no universally accepted assay or cutoff to define EBV^+^ lymphomas, with the goal of minimizing the risk of excluding patients who may benefit from this therapy.

To gain insight into the patterns and range of tumor cell EBV-positivity in this heterogeneous population, we performed postenrollment centralized EBER-ISH staining on all available tumor specimens (supplemental Table 2). There was a very broad range of positivity, with some lymphoma subtypes consistently displaying a very high percentage of EBER-ISH^+^ tumor cells (ENKTL), and others showing variable positivity (HL, AITL, and PTLD). The centralized analysis also showed that for 8 of the 33 cases of EBV^+^ lymphoma (per local laboratory), EBER-ISH could not be assessed for a variety of reasons (described in supplemental Table 3), highlighting the challenge of assessing EBER-ISH status retrospectively. In addition to technical issues, such as the lack of representative samples, tissue necrosis, and RNA degradation,^54^^,^^55^ the lineage of EBER-ISH^+^ cells often cannot be determined, as is well documented in DLBCL and AITL.^56^^,^^57^ The fact that responses, including CRs, were observed across a broad range of EBER-ISH-positivity and lymphoma types suggests that defining a cutoff as a predictive marker for response remains challenging.

EBV “viral loads” measured in plasma or whole blood at baseline or longitudinally are robust and clinically applicable prognostic biomarkers in some EBV^+^ cancers such as ENKTL^58^, ^59^, ^60^ and nasopharyngeal carcinoma,^61^ and have been studied in EBV^+^ HL,^62^^,^^63^ PTCL,^14^ and DLBCL.^64^ In ENKTL, the pEBVd is integrated into a widely used prognostic score.^65^ In ENKTL and nasopharyngeal carcinoma, pEBVd represents circulating tumor (ct) DNA, and its prognostic impact likely reflects a high tumor burden. With the possible exception of PTLD, pEBVd may not reflect EBV lytic reactivation and may therefore not be a valid predictive biomarker of the response to Nstat-VGCV. Although limited, the pEBVd data obtained in this study support published observations. First, only a fraction of patients (39/54) had elevated pEBVd at baseline, confirming that pEBVd is not a sensitive screening tool for EBV^+^ lymphoma in most subtypes. Second, decreases and increases in pEBVd levels corresponded to disease responses and progression, respectively, in several patients. Third, patients with the highest baseline levels of pEBVd were more often nonresponders and progressors (Figure 3), suggesting that a high pEBVd level may reflect a high tumor burden.

The patient cohort enrolled in this phase 1b/2 study was very heterogeneous. While safety and tolerability were good, and there was a clear efficacy signal, any conclusion about which subset of EBV^+^ lymphoma may benefit the most from this therapy would be premature. Considering that responses were observed in nearly all lymphoma subtypes, the Nstat-VGCV combination has the potential to be used in patients with most types of EBV^+^ lymphoma, and an ongoing international phase 2 study with several disease-specific cohorts (NAVAL-1, NCT05011058) is designed to generate additional data, including assessing Nstat monotherapy in PTCL. The objective responses observed in 6 patients with EBV^+^ DLBCL (2 CR, 2 PR) and in 15 patients with EBV^+^ T/NK-cell lymphomas (4 CR, 5 PR) (supplemental Table 1) and the durability of the clinical benefit (Figure 1) are highly encouraging. Although EBV^+^ DLBCL represents only 5% to 14% of all DLBCL, it is emerging as a very poor-risk subset, and the global unmet need for R/R T/NK-cell lymphomas is also very high. Therefore, the availability of an additional treatment option that is safe, effective, and orally administered may have a high impact, especially where access to care and resources is limited and EBV^+^ lymphomas are the most prevalent.

This study has limitations. First, the study design did not allow for a formal assessment of the relative contributions of Nstat and VGCV to the observed antitumor responses. Although a large body of preclinical data supports the concept that the combination of Nstat and VGCV is necessary to achieve the “kick and kill” effect in EBV^+^ tumors, we cannot exclude the possibility that some of the efficacy observed in this study may have been due to Nstat alone, because HDACi have efficacy in lymphoma. However, the number, depth, and durability of the responses observed in this heavily pretreated population suggest otherwise, particularly in DLBCL, where responses to single-agent HDACi range between 5.6% and 28%.^42^, ^43^, ^44^ The ongoing phase 2 VT3996-202 trial (NCT05011058; NAVAL-1) addresses this question in PTCL by including a single-agent Nstat cohort. Second, we did not observe a signal that a certain cutoff for EBER-ISH positivity could be explored as a biomarker for response. This is not surprising, considering the heterogeneity of the study population. We expect to gain insight into these important tumor biology questions in the near future. Finally, as no efficacy assessments were available for 12 of our 55 patients, we analyzed the effect of including patients with no efficacy assessments in the ORR calculation. In the worst-case scenario, assuming that all 12 subjects were failures, the ORR would have been 31%. However, 3 subjects either did not have EBV^+^ lymphoma (n = 2) or did not have measurable disease (n = 1) and were therefore not the target population. If the remaining subjects were conservatively included in the ORR, it would have become 33%. In fact, the 95% exact confidence interval for an ORR of 40% with n = 43 ranged from 25% to 56%, and for an ORR of 33% with n = 52 ranged from 20% to 47%. Both ranges include the current-estimated ORR of 40%.

In conclusion, targeted therapy with Nstat in combination with VGCV is a promising novel approach with the potential to fill an important gap in the complex treatment algorithm for lymphoma by addressing a recently recognized and untapped tumor-specific vulnerability shared by multiple lymphoma subtypes. Further investigation of Nstat-VGCV in the ongoing phase 2 NAVAL-1 study will further highlight the prevalence and unmet needs of patients with EBV^+^ lymphoma and the importance of routine EBV screening in aggressive lymphomas.

Conflict-of-interest disclosure: B.H. reports consultancy fees from Viracta Therapeutics. R.B. reports participation in scientific advisory boards for Viracta Therapeutics and Atara; research funding from CODIAK Biosciences; and contribution to product development for Agenus. T.A.F. declares consultancy fees or membership to the speakers’ bureaus for ADC Therapeutics, Genmab, Seattle Genetics, Secura Bio, and Takeda. E.A.B. has participated in advisory boards for ADC Therapeutics, AstraZeneca, BeiGene, Pharmacyclics/Janssen, and X4 Pharma; has participated in the speaker’s bureau for AstraZeneca, BeiGene, Incyte/MorphoSys, Pharmacyclics/Janssen, and Seattle Genetics; has been a steering committee member for Incyte/MorphoSys; and has received consultancy fees from Acrotech Biopharma. S.N. reports research funding from the Bristol Myers Squibb (BMS), 10.13039/100004334Merck Sharp & Dohme LLC, and 10.13039/100004336Novartis. P.S. has been a clinical investigator for studies by AstraZeneca, BioCryst, Novartis, Roche, and Viracta Therapeutics; has given scientific presentations for Amgen, Alexion, AstraZeneca, Novartis, Roche, and Janssen; has received grants or research support from Alnylam and 10.13039/100004319Pfizer; has received consultancy fees from AbbVie, Alexion, AstraZeneca, BioCryst, Janssen, Pfizer, and Roche; and has been a speaker for Alexion, Amgen, AstraZeneca, BMS, Novartis, and Pfizer. E.J. has received honoraria from AbbVie, BeiGene, and Takeda, and has participated in advisory boards for AstraZeneca and Epizyme. D.V.F. has received consultancy fees from Viracta Therapeutics; holds stock in Viracta Therapeutics; discloses employment and equity in Phoenicia Biosciences, Inc and Oryzon Genomics, Inc; equity in Takeda Pharmaceuticals and Briacell Therapeutics, Inc; and consulting fees from Molecular Partners, Inc and Briacell Therapeutics, Inc. P.P. has received research funding from Viracta Therapeutics, Inc and 10.13039/100006259Teva Pharmaceuticals; honoraria from Viracta Therapeutics, Inc, Innate Pharma, Daiichi, Kyowa, Teva Pharmaceuticals, and DrenBio; has participated in scientific advisory boards for Viracta Therapeutics, Inc, Innate Pharma, BeiGene, Kyowa, MorphoSys, ADCT, Loxo, and ONO Pharmaceutical; and has received consultancy fees from DrenBio and Viracta Therapeutics, Inc. A.K., I.R., and L.R. are employed by Viracta Therapeutics, Inc, Cardiff, CA. I.R. serves on the Board of Directors for and holds stock in Viracta Therapeutics, Inc, Cardiff, CA. R.M. and D.V.F. were employed by Viracta Therapeutics, Inc, Cardiff, CA at the time of this study. The remaining authors declare no competing financial interests.

The current affiliation for D.V.F. is Oryzon Genomics, Boston, MA.
