# Supplementary material for: Targeted therapy with nanatinostat and valganciclovir in recurrent EBV-positive lymphoid malignancies: a phase 1b/2 study
Source: Blood Adv. 2023 Aug 4;7(20):6339–50. doi: 10.1182/bloodadvances.2023010330 (PMC10587711; doi:10.1182/bloodadvances.2023010330)
Supplement: Supplemental Methods, Tables, and Figure [file BLOODA_ADV-2023-010330-mmc1.pdf]

SUPPLEMENTARY DATA

Supplementary Methods

1. Eligibility criteria

Inclusion criteria

1. Signed, informed consent
2. Age 18 or more years
3. ECOG PS26 of 0 to 2 or Karnofsky Performance scale (KPS) ≥ 60%
4. Failed any available standard therapy with a reasonable likelihood of clinical benefit (e.g., anti-CD20 monoclonal antibody, withdrawal of immunosuppression, cytotoxic chemotherapy)
5. Relapsed/refractory, pathologically confirmed EBV+ lymphoid malignancy or lymphoproliferative disease regardless of histologic subtype
6. EBV+ as determined by institution's usual testing method
7. Absence of available therapy with reasonable likelihood of cure or significant clinical benefit
8. Evaluable [Phase 1b only] or measurable [Phase 1b and Phase 2] disease based on Recommendations for Initial Evaluation, Staging, and Response Assessment of Hodgkin and Non-Hodgkin Lymphoma: The Lugano Classification (Cheson BD, Fisher RI, Barrington SF, et al. Recommendations for Initial Evaluation, Staging, and Response Assessment of Hodgkin and Non-Hodgkin Lymphoma: The Lugano Classification. J Clin Oncol. 2014;32(27):3059-3067.)
9. If previously treated:
    a. Treatment-related toxicity resolved to at least Grade 1 (alopecia excepted), or
    b. Treatment-related toxicity resolved to at least Grade 2 with prior approval of the Medical Monitor, or
    c. Treatment related toxicity resolved to at least the levels outlined in inclusion criterion #10
10. Adequate laboratory parameters including:
    a. Absolute neutrophil count (ANC) ≥ $1000/mm^3$
    b. Platelet (PLT) count ≥ $50,000/mm^3$
    c. Asparagine aminotransferase (AST)/serum glutamine oxaloacetic transaminase (SGOT), alanine aminotransferase (ALT)/serum glutamic pyruvic transaminase (SGPT) ≤3.0 x upper limit of normal (ULN) (≤5 xULN if known liver involvement)
    d. Total bilirubin ≤2.0 x ULN unless considered due to Gilbert's syndrome in which case, ≤3.5 x ULN

e. Estimated glomerular filtration rate (eGFR) ≥60 mL/min/1.732 m^2 by Modification of Diet in Renal Disease (MDRD) equation
f. Prothrombin time (PT) ≤1.5 x ULN
g. International normalized ratio (INR) ≤1.5 x ULN
h. Serum potassium and magnesium should be within normal limits for institution or treatment to correct out of range values should be instituted

11. Willingness to participate in collection of all required laboratory testing as defined in the protocol
12. Females must be surgically sterile, postmenopausal, or agree to use adequate contraception (adequate as determined by the judgement of the Investigator) throughout the study and for a period of 6 months after last dose of nanatinostat
13. Males must be surgically sterile or must agree to use of effective contraception throughout the study and for a period of 3 months after last dose of nanatinostat

Exclusion criteria

1. Fewer than 14 days from prior chemotherapy, irradiation, biological, or investigational therapy (fewer than 7 days from prior therapies if approved by the Medical Monitor)
2. Fewer than 28 days from receipt of prior HDAC inhibitor
3. Fewer than 60 days from prior hematopoietic stem cell transplantation or solid organ transplantation
4. Known primary CNS lymphoma
5. CNS metastases or leptomeningeal disease unless appropriately treated and neurologically stable for at least 4 weeks
6. Other known active cancer(s) likely to require treatment in the next year that would impact the assessment of any study endpoints
7. Refractory graft versus host disease (GvHD) not responding to treatment (e.g., steroid refractory and not responding to second-line agents)
8. Active, uncontrolled bacterial, viral, or fungal infection(s) requiring systemic therapy
9. Pregnant or currently breastfeeding
10. Positive hepatitis B core antibody or surface antigen unless quantitative DNA PCR is negative and patient will be receiving prophylaxis for reactivation
11. Positive hepatitis C virus on PCR
12. History of allergic reactions attributed to compounds of similar chemical or biologic composition to valganciclovir
13. Psychiatric illness/social situations that would interfere with compliance with study requirements
14. Clinically significant cardiovascular abnormalities such as uncontrolled hypertension, congestive heart failure (New York Heart Association classification ≥2), unstable angina, poorly controlled arrhythmias, or myocardial infarction within 6 months of

study entry. Congenital long QT syndrome or QTcF interval of ≥ 470 msec, average of triplicate readings at screening

15. Other severe acute or chronic medical or psychiatric conditions or laboratory abnormalities that would impart, in the judgement of the Investigator, Medical Monitor, and/or Sponsor representative, excess risk associated with study participation or would interfere with study outcome assessments
16. Known involvement of critical structures by lymphoma that is considered highly likely to result in serious outcome in the event of rapid tumor destruction
17. Known history of HHV-6 chromosomal integration (ciHHV-6)
18. Known history of HIV infection

2. Definition of dose-limiting toxicities (DLTs)

DLTs were defined as the following events occurring during Cycle 1 and not related to the underlying disease: Grade 4 anemia, febrile neutropenia, neutropenia lasting >5 days, any other Grade 4 hematologic event or Grade ≥4 tumor lysis syndrome; Grade ≥3 thrombocytopenia with/without bleeding, any requirement for platelet transfusion, any Grade ≥3 non-hematologic event despite adequate supportive care, or any event requiring a dose hold of >7 consecutive days.

3. Phase 2 sample size calculation

Sample size for Phase 2 was based on a Simon's 2-Stage design where the Phase 2 portion of the study may be stopped if none of the first 10 treated patients have a response. For a heterogenous population of disease subtypes with potentially different responses to therapy, the hypothesized response rate for a poor drug was estimated to be 5%, while the hypothesized response rate of a good drug would be at least 20%. Using a 1-sided alpha of 0.05 and power of 80%, if at least 1 of the first 10 patients has a response, the Simon's design recommends accruing up to at least 29 total subjects to test the null hypothesis. The probability of stopping early is 59.9% with a type I error rate of 0.0468.

Supplementary Results

1. RP2D dose selection

The first four patients enrolled into Cohort 1 received Nstat 10 mg PO BID + VGCV 900 mg PO BID. Three patients developed DLTs (1 Grade 4 neutropenia lasting 29 days, 1 Grade 3 thrombocytopenia lasting 6 days, and 1 Grade 4 thrombocytopenia lasting 8 days); 3/4 patients required dose interruptions lasting >7 days. This dosing was considered to have exceeded the MTD and the dose of VGCV was reduced to 450 mg BID. Three more patients were subsequently enrolled into Cohort 1, of whom one reported a DLT (Grade 3 thrombocytopenia lasting 7 days). No DLTs were reported in Cohorts 2a, b or c. After review of the Cohort 1 and 2 data by the SRC, the protocol was amended to include an additional dose level (Cohort 3): Nstat 20 mg QD "4 days on, 3 days off" with VGCV 900 mg QD. Since one DLT occurred in the 3 patients enrolled in Cohort 1 receiving Nstat 10 mg BID with VGCV 450 mg BID, introducing an interrupted schedule for Nstat in Cohort 3 was recommended by the SRC. This decision to investigate the 4 days on / 3 days off regimen was also based on several factors from published literature and from preclinical studies with Nstat:

- Preclinical studies demonstrated that discontinuous exposure to the HDACi butyrate was sufficient to sensitize P3HR1 cells (EBV+ Burkitt's lymphoma) to GCV, introducing an opportunity to minimize potential toxicities while maintaining efficacy in clinical regimens (Ghosh SK, Forman LW, Akinsheye I, Perrine SP, Faller D V. Short, discontinuous exposure to butyrate effectively sensitizes latently EBV-infected lymphoma cells to nucleoside analogue antiviral agents. Blood Cells, Mol Dis. 2007;38(1):57-65).

- Dose-response studies of HDACi, including Nstat, indicate that the levels and duration of viral PK gene induction are dose-dependent. *In vivo*, a single dose of Nstat 20 mg achieved a higher Cmax vs two 10 mg doses separated by several hours.

Supplementary Table 1. Prior therapies and responses to study treatment in evaluable patients

Age, sex	Subtype	Lines of prior therapy + responses (in chronological order)	Refractory to last therapy	Best response on study	Response duration at primary analysis (m)	Response duration at updated analysis (m)
**Evaluable patients with T/NK-NHL (n=15)**						
60, F	ENKTL	IMEP +PEG **(CR)**; GDP **(PD)**; nivolumab + EBV-specific T-cells **(PD)**; EBV-specific T-cells **(PD)**	Y	**CR**	3.8	3.8
37, F	ENKTL	SMILE **(PD);** autoSCT	Y	**PR**	26.5*	35.6*
48, M	ENKTL	SMILE **(CR)**; SMILE **(CR)**; cisplatin + XRT **(PD)**	Y	**PR**	3.6	3.6*
44, M	ENKTL	M-SMILE **(CR)**; autoSCT **(CR)**; pembrolizumab **(PD)**	Y	**PR**	2.7	2.7
47, F	ENKTL	CHOP **(PR)**; CHOP **(PD)**; mSMILE **(PD)**; alloSCT; ATA-129 **(PD)**; pembrolizumab **(PD)**	Y	**PR**	7.5*	16.8*
76, F	ENKTL	GEMOX **(PD)** ; XRT **(SD)**; pembrolizumab **(SD)**	Y	PD	–	–
47, F	ENKTL	SMILE **(PD)**	Y	PD	–	–
69, F	ENKTL	P-GEMOX (N/A)	Y	PD	–	–
63, M	AITL	CHOP **(CR)**; ICE **(PD)**	Y	**CR**	32.2*[†]	-
66, M	AITL	CHOP **(PR)**	Y	**CR**	15.4*	24.5*
78, M	AITL	CHOP **(CR)**; autoSCT; prednisone **(PD)**	Y	SD	–	–
58, M	PTCL NOS	CHOEP **(CR)**; romidepsin **(CR)**; autoSCT; romidepsin ("no response"); XRT **(SD)**	Y	PR	10.8	10.8
74, F	PTCL NOS	CHOP **(PR)**; romidepsin **(PD)**	Y	**CR**	5.8	5.8
71, F	PTCL NOS	CHOP **(CR)**; brentuximab **(PD)**; ICE **(PD)**	Y	PD	–	–
43, M	CTCL	Targretin **(PD)**; extracorporeal photopheresis **(SD)**; alloSCT **(CR)**, alemtuzumab **(CR)**; Resimmune **(PD)**; bexarotene + UVB **(PD)**; vorinostat **(PD)**; brentuximab **(PD)**	Y	PD	–	–

Evaluable patients with EBV+ DLBCL, NOS (n=6)

		Lines of prior therapy + responses (in chronological order)	Past medical history				
77, M	DLBCL	R-EPOCH **(CR)**; R-GDP (discontinued due to toxicity); PBR **(PD)**	HTN, rheumatoid arthritis (RA), atrial fibrillation (a fib) (a fib)	No	**PR**	14.8*	30.3*
76, F[‡]	DLBCL	R-CHOP **(CR x 2 m)**	Interstitial lung disease, peripheral neuropathy	Yes	**CR**	1.9	1.9
80, M	DLBCL	R-CHOP **(CR)**	ECOG 2, interstitial pulmonary	No	**PR**	4.0	4.0

			fibrosis, RA, COPD (O2- dependent), peripheral neuropathy				
67, M[‡]	DLBCL	R-CHOP **(PD)**	Hospitalized with febrile neutropenia during CHOP Cycle 7	Yes	**CR**	20.3*	35.0*
76, M	DLBCL	Radiotherapy, R-CHOP **(CR)**	Essential tremor, depression	Yes	PD	N/A	N/A
78, M	DLBCL	Antecedent history of EBV$^+$AITL (with 5 lines of therapy)	Prior CHOP, ICE, anti-ICOS ab, otlertuzumab + bendamustine	N/A	SD	N/A – w/d on study day 78	N/A (w/d on study day 78)

*Response ongoing; [†]patient in CR 20.7 m after stopping therapy; [‡]primary refractory.

AITL, angioimmunoblastic T-cell lymphoma; alloSCT, allogeneic stem cell transplantation; ASCT, autologous stem cell transplantation; autoSCT, autologous stem cell transplantation; BEAM, carmustine, etoposide, cytarabine, melphalan; CHOP, cyclophosphamide, doxorubicin, vincristine, prednisone; CHOEP, cyclophosphamide, doxorubicin, vincristine, etoposide, prednisone; CTCL, cutaneous T-cell lymphoma; CR, complete response; DLBCL, diffuse large B-cell lymphoma; EBV, Epstein-Barr virus; ENKTL, extranodal natural killer/T-cell lymphoma; GDP, gemcitabine, dexamethasone, cisplatin; F, female; GemOx, gemcitabine, oxaliplatin; ICE, ifosfamide, carboplatin, etoposide; IFRT, involved-field radiation therapy; IMEP, ifosfamide, methotrexate, etoposide, prednisone; m, months; M, male; mSMILE, modified SMILE regimen; N/A, not applicable; PBR, polatuzumab vedotin, bendamustine, rituximab; P-GEMOX, pegaspargase, gemcitabine, oxaliplatin; PR, partial response; PTCL NOS, peripheral T-cell lymphoma not otherwise specified; R-CHOP, rituximab, cyclophosphamide, doxorubicin, vincristine and prednisone; R-EPOCH, rituximab, etoposide phosphate, prednisone, vincristine, cyclophosphamide, and doxorubicin; R-GDP, rituximab, gemcitabine, cisplatin, and dexamethasone; SD, stable disease ; SMILE, dexamethasone, methotrexate, ifosfamide, L-asparaginase, etoposide; R, rituximab; w/d, withdrawn; XRT, radiation therapy.

Supplementary Table 2. Lymphoma subtype, response and EBER-ISH positivity

Lymphoma subtype	Response (Investigator-assessed)	Local method used to determine EBV+ at enrollment	% EBER-ISH assessment* (post-enrollment central analysis)
**<10% EBER-ISH positivity**			
DLBCL	CR	EBER-ISH	<1
AITL	CR	EBER-ISH	<1
PTCL NOS	PR	EBER-ISH	1
AITL	SD	EBER-ISH	1
Hodgkin	SD	LMP-1	1
Hodgkin	PD	EBER-ISH	1
Hodgkin	SD	EBER-ISH	2
B-LPD	PD	EBER-ISH	3
Hodgkin	PR	EBER-ISH	3
DLBCL	SD	EBER-ISH	5
Hodgkin	PD	EBER-ISH, LMP-1	8
**10–50% EBER-ISH positivity**			
PTCL NOS	CR	EBER-ISH	10
AITL	CR	EBER-ISH	10
Hodgkin	SD	EBER-ISH	15
B-LPD	PD	EBER-ISH	15
IA-LPD	PD	EBER-ISH	25
Hodgkin	SD	EBER-ISH	30
HIV-L	PD	EBER-ISH	40
**>50% EBER-ISH positivity**			
ENKTL	PR	EBER-ISH	60
PTLD	PD	EBER-ISH	70
DLBCL	PR	EBER-ISH	70
ENKTL	PR	EBER-ISH	70
HIV-L	PD	EBER-ISH	70
ENKTL	PD	EBER-ISH	80
ENKTL	PD	EBER-ISH	80

* To be considered evaluable, 100 viable tumor cells per H&E-stained slide must be present.
% EBER-ISH positivity = % EBER-ISH+ cells /all viable tumor cells.

AITL, angioimmunoblastic T-cell lymphoma; B-LPD, B-cell lymphoproliferative disease; CTCL, cutaneous T-cell lymphoma; DLBCL, diffuse large B-cell lymphoma; EBER-ISH, EBV-encoded RNA *in situ* hybridization; ENKTL, extranodal natural killer/T-cell lymphoma; H&E, hematoxylin and eosin; HIV-L, HIV-associated lymphoma; IA-LPD, immunodeficiency-associated lymphoproliferative disorders; LMP-1, latent membrane protein-1; PTCL NOS, peripheral T-cell lymphoma not otherwise specified; PTLD, post-transplant lymphoproliferative disorder.

Supplementary Table 3. Technical issues for the Eight EBER-ISH Samples Reported Negative by Central Laboratory Testing

No	Lymphoma subtype	Response	Method used to determine EBV+ at site	% EBER-ISH positivity (NG*)	Explanation
1	PTLD	PD	EBER-ISH	0	Biopsy sample was 5 years old (2014)
2	IA-LPD	PR	LMP-1	0	EBV positivity was determined using LMP-1 at site
3	PTLD	CR	EBER-ISH	0	Not determined
4	DLBCL	PR	EBER-ISH	0	Specimen collected is less likely representative of tumor tissue
5	IA-LPD	CR	EBER-ISH	0	Lymph node fragments: “small subset of large cells expressing EBER” - suboptimal biopsy
6	Hodgkin	SD	EBER-ISH	0	“Extensive necrosis” reported for biopsy sample
7	CTCL	PD	EBER-ISH	0	CTCL is infrequently associated with EBV* - Extremely rare EBER positive cells were detected; unclear whether EBV+ cells are tumor cells
8	PTCL, NOS	PD	EBER-ISH	0	A pre-treatment (prior to 1L) tumor biopsy from July 2019 reported “scattered EBER +” cells (origin not mentioned). Sample was reviewed by NG and reported negative. Patient subsequently had 2 relapses with additional therapies and EBER-ish was not mentioned in the path reports from the more recent biopsy.

*Pan ST, Chang WS, Murphy M, Martinez A, Chuang SS. Cutaneous peripheral T-cell lymphoma of cytotoxic phenotype mimicking extranodal NK/T-cell lymphoma. Am J Dermatopathol. 2011 Apr;33(2):e17-20. doi: 10.1097/DAD.0b013e3181ea6571

CTCL, cutaneous T-cell lymphoma; DLBCL, diffuse large B-cell lymphoma; EBER-ISH, EBV-encoded RNA *in situ* hybridization; IA-LPD, immunodeficiency-associated lymphoproliferative disorders; LMP-1, latent membrane protein-1; NG, Sponsor’s Central Laboratory (NeoGenomics, FL, USA); PTCL NOS, peripheral T-cell lymphoma not otherwise specified; PTLD, post-transplant lymphoproliferative disorder.

Supplementary Figure 1. Patient flow chart

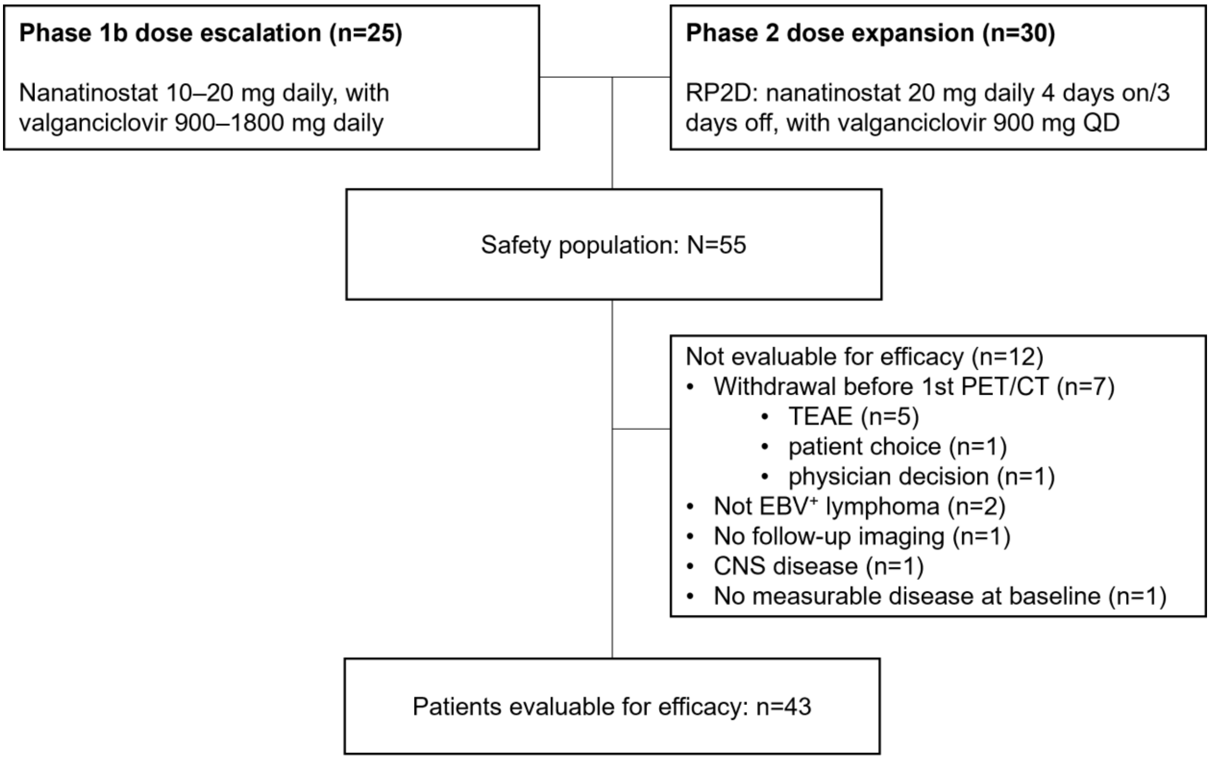

CNS, central nervous system; EBV, Epstein-Barr virus; PET/CT, positron emission tomography/computed tomography; RP2D, recommended Phase 2 dose; TEAEs, treatment-emergent adverse events
